# Daily Timed Sexual Interaction Induces Moderate Anticipatory Activity in Mice

**DOI:** 10.1371/journal.pone.0015429

**Published:** 2010-11-03

**Authors:** Cynthia T. Hsu, Piotr Dollár, Daniel Chang, Andrew D. Steele

**Affiliations:** 1 Broad Fellows Program in Brain Circuitry, Division of Biology, California Institute of Technology, Pasadena, California, United States of America; 2 Division of Engineering and Applied Science, California Institute of Technology, Pasadena, California, United States of America; Vanderbilt University, United States of America

## Abstract

Anticipation of resource availability is a vital skill yet it is poorly understood in terms of neuronal circuitry. Rodents display robust anticipatory activity in the several hours preceding timed daily access to food when access is limited to a short temporal duration. We tested whether this anticipatory behavior could be generalized to timed daily social interaction by examining if singly housed male mice could anticipate either a daily novel female or a familiar female. We observed that anticipatory activity was moderate under both conditions, although both a novel female partner and sexual experience are moderate contributing factors to increasing anticipatory activity. In contrast, restricted access to running wheels did not produce any anticipatory activity, suggesting that an increase in activity during the scheduled access time was not sufficient to induce anticipation. To tease apart social versus sexual interaction, we tested the effect of exposing singly housed female mice to a familiar companion female mouse daily. The female mice did not show anticipatory activity for restricted female access, despite a large amount of social interaction, suggesting that daily timed social interaction between mice of the same gender is insufficient to induce anticipatory activity. Our study demonstrates that male mice will show anticipatory activity, albeit inconsistently, for a daily timed sexual encounter.

## Introduction

The ability to anticipate events is crucial for survival. In particular, timing activity to take advantage of resource availability helps optimize energy usage and avoid predation. The most well-studied model of this phenomenon is food anticipatory activity (FAA), in which temporally restricting the availability of food results in an increase in activity prior to the time of feeding [1]–[2]. Surprisingly, the principle neural structure regulating circadian rhythms, the suprachiasmatic nucleus, is not required for FAA [3]. Attempts to determine the neural circuit mediating FAA have not yet converged on a common target, despite numerous lesion studies [4]–[5]. Genetic approaches in mice have recently implicated hunger and arousal hormones/peptides in mediating FAA [6]–[10], furthering the notion that hypothalamic circuitry is critically involved in mediating anticipatory activity (AA).

Since daily restricted water access [11]–[13] and palatable meal access in rats [14]–[17] can also induce moderate AA, it is a relevant to ask whether FAA is a specific “food-seeking behavior” [16]. Another possibility is that FAA represents one of several appetitive drives in the rodent, such that limited but scheduled daily access to the motivating stimulus will lead to AA. We reasoned that sexual activity or even companionship for a singly housed mouse could also provide form of daily pleasure. To test this hypothesis, we gave C57BL/6J male mice restricted access to female mice for one hour daily. We quantified their home cage behaviors by weekly video recording in the home cage using a computer vision system to quantify activity. We observed a variable AA response in males in two different restricted female access conditions, whereas restricted access to a running wheel did not lead to AA. Daily timed interaction between two female mice did not lead to AA, suggesting that the male mice that did show AA for restricted female access did so due to anticipation of a sexual, as opposed to a purely social, encounter.

## Results

### Restricted Social Interaction Experiment 1

To test for the ability of male mice to anticipate novel social interaction, a group of C57BL/6J male mice (n = 12) were divided into two groups: the experimental group (n = 6) received restricted access to a novel female for an hour each day during the light cycle from Zeitgeber Time (ZT) 9 to ZT 10 (13L: 11D cycle; by convention ZT 12 is “lights off”) while the control group (n = 6) was disturbed slightly at both ZT 9 and ZT 10 to control for handling artifacts. To monitor activity levels, these mice were video recorded in their home cages for 23 h, starting at ZT10 (only while single housed) at days 0, 7, 14, 21, and 28 of this treatment. The videos were analyzed by an automated behavior recognition system, HomeCageScan 3.0 [18]–[19], which quantifies behaviors such as food bin entry, drinking, hanging, jumping, rearing, walking, grooming, and several others.

**Figure 1:**
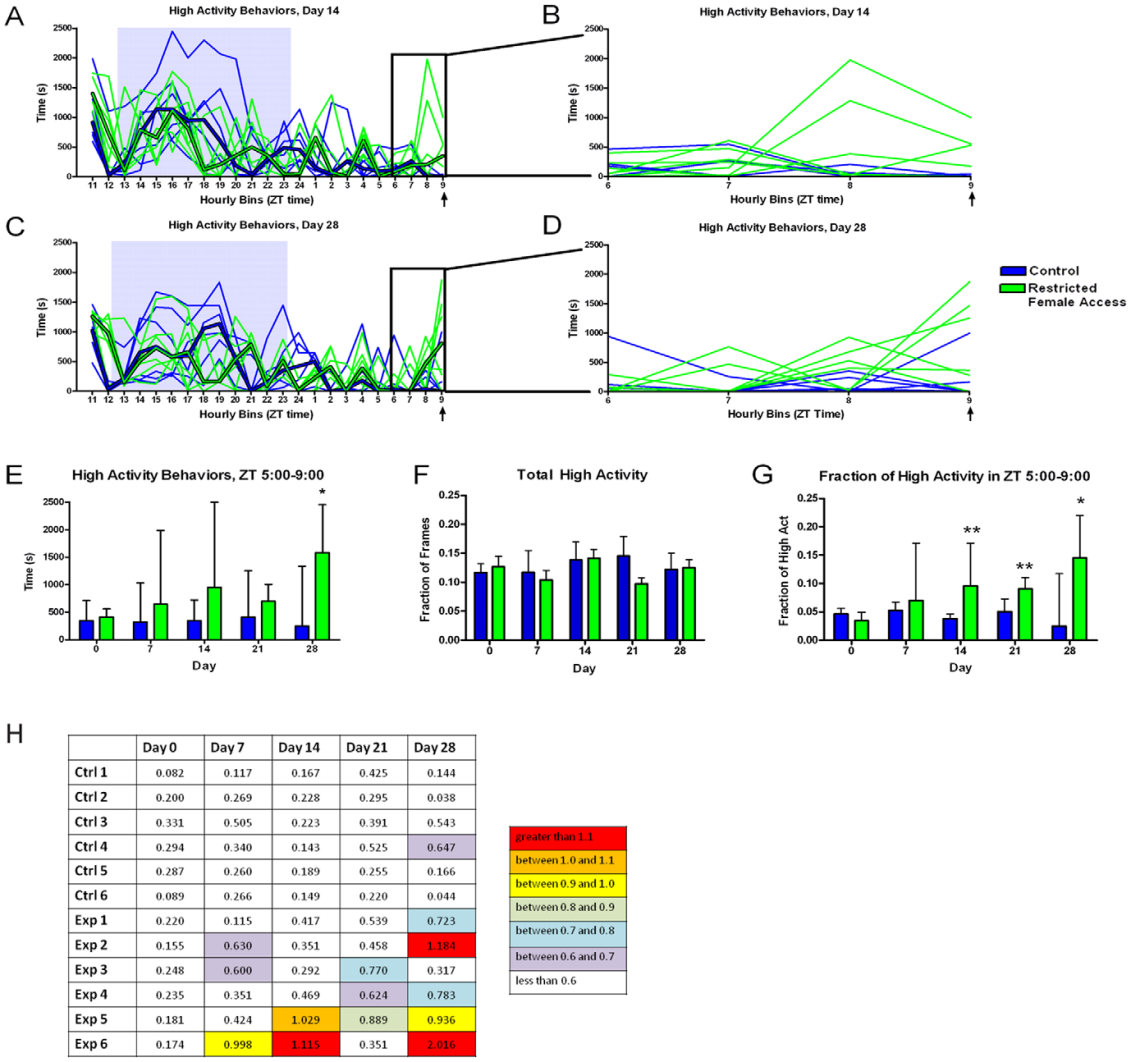
Characterizing the anticipatory activity in the home cage of male mice with one hour of restricted access to a novel female daily (“Experiment #1”). (**A**) The number of seconds in each hourly bin of high activity behaviors (walking, jumping, rearing, and hanging) that occurred on day 14 of restricted female access. Thin weight green lines represent data from individual mice with restricted female access while blue lines represent the controls. Median values are shown in thick green and blue lines for experimental and control mice, respectively. The gray area depicts the 11 hours of lights off. (**B**) An enlarged presentation of ZT 5 to 9, the four hours preceding female access, in panel A, to show the variance in the anticipatory behavior. (**C**) The number of seconds in each hourly bin of high activity behaviors that occurred on day 28 of the experiment. (**D**) An enlarged representation of bins 6 to 9 (which includes data from ZT 5-9) in C. (**E**) The sum of the number of seconds spent in any high activity behavior (walking, jumping, rearing, and hanging) during the last four hours prior to restricted female access. (**F**) Fraction of frames during the entire duration of the recording (∼23 hrs) during which the mice exhibited any high activity behavior. (**G**) Normalized high activity data in the four hours preceding restricted female access. The number of seconds spent in high activity behavior during the last four hours prior to female access divided by the total number of seconds of high activity across the entire 23 hour recording. (**H**) Table illustrating the relative strength of anticipatory activity versus nighttime activity for each individual mouse at every measurement in the experiment. For each animal, the maximum number of seconds per hour the mouse exhibits between ZT 5 and 9 is divided by the maximum number of seconds per hour the mouse exhibits during the lights off period. A ratio of greater than 1.1 is highlighted in red, between 1.0 and 1.1 in orange, between 0.9 and 1.0 in yellow, between 0.8 and 0.9 in light green, 0.7 and 0.8 in blue, 0.6 and 0.7 in purple, and less than 0.6 is white. All bar graphs represent median and the upper quartile of the interquartile range. N = 6 at all time points. Mann-Whitney Test, * = p<0.05, ** = p<0.01, *** = p<0.001.

We examined the temporal aspects of home-cage activity to determine whether male mice show an increased activity (defined as the summation of time spent rearing, walking, hanging, and jumping) in the hours prior to daily female access (Figure 1A,C). We noted that some mice increased activity prior to the time of restricted female access but others did not (Figure 1A-B, H). For example, after 14 days of restricted female access, only two mice (out of 6 total) showed an increase in activity in the hours preceding female access comparable to the nighttime activity peak (Figure 1A, 1B). On day 28 of the experiment, three mice (out of 6 total) showed an increase in activity comparable to the nighttime activity peak (Figure 1C, 1D). Interestingly, the individual mice that showed AA did not do so consistently from week-to-week (Fig. 1H). Summing the amount of time occupied by high activity behaviors over hours ZT 5-9 (just prior to female access) across each measurement showed a trend toward increased activity at several time points, particularly in the upper quartile range, but the only significant increase in activity occurred on day 28 (Mann-Whitney Test, p<0.05) (Figure 1E).

We next asked whether the one hour of daily female exposure influenced the amount of total activity (i.e., in the remaining 23 hours). There were no significant differences in the overall activity between mice with restricted female access and control mice (Mann-Whitney Test) (Figure 1F). Because this comparison does not account for a change in the distribution of activity, we next asked the question—“How much of the high activity behaviors occurred in the hours preceding female access relative to the total activity?” Interestingly, when the seconds of activity during the four hours prior to female access was divided by the seconds of total high activity, there was a significantly greater fraction of AA in restricted female access mice than in controls at several time points, days 14, 21, and 28, suggesting that male mice can anticipate the arrival of a female mouse by showing increased activity prior to receiving the female relative to their overall activity (Mann-Whitney Test, p<0.05) (Figure 1G).

### Restricted Social Interaction Experiment 2

We repeated the timed female access experiment but changed two parameters to test how anticipation of social interaction is influenced by 1) mating experience and 2) familiar partners. First, both the experimental (n = 8) and control (n = 8) groups of male mice were cohabitated with a female for seven days (from day -7 until day 0). Second, starting on day 0, each mouse in the experimental group was paired daily with the same female for one hour for an entire week. At the end of the week of exposure to the same female for one hour the mice were video recorded to measure their activity. The male was assigned to a new, virgin female at the end of the weekly recording, and this procedure was repeated for five weeks total (with recordings starting at day -7 in this experiment). Control mice were handled in parallel with experimental mice but not exposed to females with the exception of the initial cohabitation period during day -7 to day 0 of the experiment.

**Figure 2:**
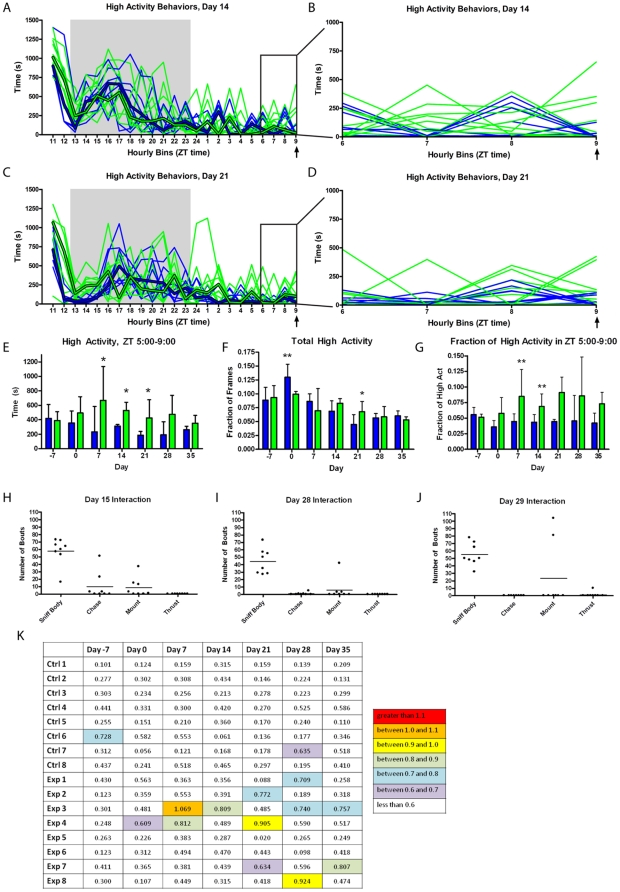
Characterizing the anticipatory activity in the home cage of male mice with one hour of restricted access to a female partner daily (the same female for seven consecutive days, “Experiment #2”). (**A**) The number of seconds in each hourly bin of high activity behaviors (walking, jumping, rearing, and hanging) that occurred on day 14 of restricted female access. Thin weight green lines represent data from individual mice with restricted female access while blue lines represent the controls. Median values are shown in thick green and blue lines for experimental and control mice, respectively. The gray area depicts the 11 hours of lights off. (**B**) An enlarged presentation of ZT 5 to 9, the four hours preceding female access, in panel A, to show the variance in the anticipatory behavior. (**C**) The number of seconds in each hourly bin of high activity behaviors that occurred on day 21 of the experiment. (**D**) An enlarged representation of bins 6 to 9 (which includes data from ZT 5-9) in C. (**E**) The number of seconds in each hourly bin of high activity behaviors (walking, jumping, rearing, and hanging) during the last four hours prior to restricted female access. (**F**) Fraction of frames during the entire duration of the video recording (∼23 hrs) during which the mice exhibited any high activity behavior. (**G**) Normalized high activity data in the four hours preceding restricted female access. The number of seconds spent in high activity behavior during the last four hours prior to female access divided by the total number of seconds of high activity across the entire 23 hour recording. Quantifying the social interaction during the restricted female access period. The number of bouts in which each male mouse interacted with its respective female by sniffing the body, chasing, mounting, or thrusting on (**H**) day 15 is displayed as a scatter plot, with the solid line indicating the median value. (**I**) Sexual/social interaction on day 28 with the same female partner for the previous six days. (**J**) Interaction on day 29 with a novel female. (**K**) Table illustrating the relative strength of anticipatory activity versus nighttime activity for each individual mouse at every measurement in the experiment. For each animal, the maximum number of seconds per hour the mouse exhibits between ZT 5 and 9 is divided by the maximum number of seconds per hour the mouse exhibits during the lights off period. A ratio of greater than 1.1 is highlighted in red, between 1.0 and 1.1 in orange, between 0.9 and 1.0 in yellow, between 0.8 and 0.9 in light green, 0.7 and 0.8 in blue, 0.6 and 0.7 in purple, and less than 0.6 is white. n = 8 at all time points. All bar graphs represent median and the upper quartile of the interquartile range. * = p<0.05, ** = p<0.01, *** = p<0.001, Mann-Whitney Test.

We examined the temporal aspects of home-cage activity to determine whether male mice show an increased activity in the hours preceding to daily female access (Figure 2A-D). We noted that some mice increased activity prior to the time of restricted female access but others did not at days 14 and 21 of restricted female access (Figure 2B,D, and K). The total number of seconds of high activity during the four hours prior (ZT 5-9) to female access was significantly higher for the mice with restricted female access than for the controls on days 7, 14, and 21 (Mann-Whitney Test, p<0.05) (Figure 2E). There were no consistent differences in global activity with the exception of day 0 where the control group showed an increased overall activity and day 21 where the males with female access showed a slight increase in total activity (Mann-Whitney Test, p<0.05) (Figure 2F). To normalize for total activity levels, we took the amount of time spent in activity during the four hours prior to female access and divided by the total amount of time spent in high activity behaviors. The fraction of high activity during the four hours prior to restricted female access was only significantly greater than the control mice at days 7 and 14 (Mann-Whitney Test, p<0.01), although there are trends toward increased activity in the upper quartile of mice at days 21, 28, and 35 (Figure 2G), again showing the same phenomenon that we described in ‘restricted social interaction experiment 1’ where only a portion of males show increased AA.

In order to determine the amount of social and sexual interaction during the restricted female access, we video recorded the hour of female access on days 15, 28, and 29. Days 15 and 29 were with novel female partners, while day 28 was interaction with a female with which the mice had been partnered repeatedly with for seven days in a row. This enabled us to assess if males interact with novel females differently than they interact with females they have grown accustomed to. Interaction between the male and female mice was quantified by manual annotation of video recordings in which the behaviors were categorized as sniff body, approach, chase, walk away, mount and attempt mount, thrust and/or ejaculation, or non-interacting. The male mice were awake during the entire one hour period (data not shown). Most of the interaction between the males and females was exhibited in the form of sniffing: the males sniffed the female a median of 62 times on day 15, 42.5 times on day 28, and 50 times on day 29 (Figure 2H-J). The differences in the number of bouts of sniffing were not statistically significant between the three days that this behavior was quantified (Kruskal-Wallis Test). Overall, the profile of mating and interacting behaviors was similar at days 15, 28, and 29. However, two of the males interacting with a novel female (day 29) exhibited almost twice as many instances of mount and attempted mount (81 and 104) as mice did on days 15 (at most 37 bouts) and 28 (at most 42 bouts), when interacting with the same females they had received the previous seven days (Figure 2J). Ejaculation was only observed in one individual in day 28 and one individual on day 29, suggesting that actual copulatory events were rare during the one hour of daily social interaction.

### Analysis of Eating Behavior

Restricted feeding is the most well-documented example of a behavioral manipulation that induces AA, and self-imposed changes in the timing of food intake have been shown to contribute to the AA observed in rats limited to 1 h of water access each day, with free access to food [11]. To determine if the mild AA that we observed was the consequence of self-imposed food restriction, we examined the temporal distribution of food bin entries output by the HomeCageScan software.

**Figure 3:**
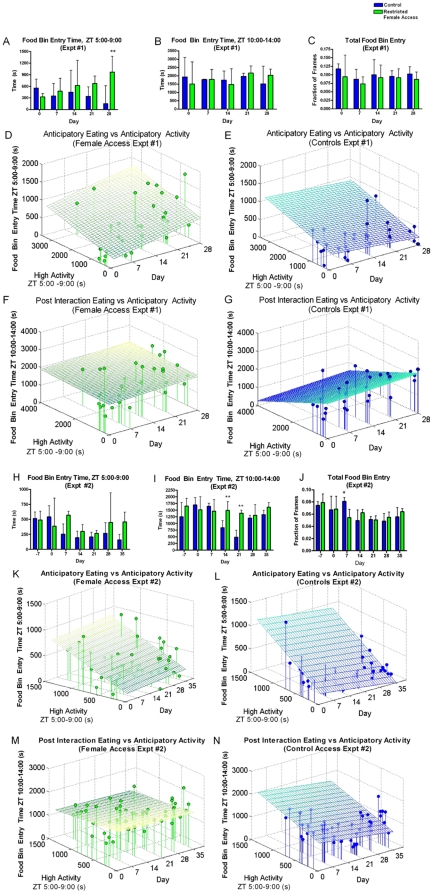
Examination of eating behavior in mice with restricted female access. (**A**) For social experiment #1, the sum of the number of seconds control mice (dark blue) enter the food bin during the four hours prior to the interaction time and the number of seconds that mice with restricted female access (green) spent entering the food bin during the four hours prior to the interaction time. (**B**) For social experiment 1, the sum of the number of seconds mice enter the food bin during the four hours after the interaction time. (**C**) For social experiment 1, the fraction of frames during the entire duration of the recording in which the mice showed food bin entry. Analysis of correlations between eating and activity across experiments #1 and #2. (**D**) The total number of seconds that each restricted female access mouse spends entering the food bin in the four hours before female access (ZT 5-9) is plotted against the number of seconds of high activity in the four hours before female access (ZT 5-9) and the time point in the experiment (days 0, 7, 14, 21, and 28). The best-fit plane is shown (R = 0.6609, p = 0.0004). (**E**) The total number of seconds that each control mouse spends entering the food bin in the four hours before feeding (ZT 5-9) is plotted against the number of seconds of high activity in the four hours before feeding (ZT 5-9) with the best-fit plane shown (R = 0.3688, p = 0.0960). (**F**) The total number of seconds that each restricted female access mouse spends entering the food bin in the four hours after female access is plotted against the number of seconds of high activity in the four hours before female access and the time point in the experiment with the best-fit plane shown (R = 0.1208, p = 0.8201). (**G**) The total number of seconds that each control mouse spends entering the food bin in the four hours after feeding is plotted against the number of seconds of high activity in the four hours before feeding across the experiment (R = 0.3282, p = 0.2146). (**H**) For social experiment #2, the sum of the number of seconds control mice enter the food bin during the four hours prior to the interaction time. (**I**) For social experiment #2, the sum of the number of seconds mice enter the food bin during the four hours after the interaction time and (**J**) represents the fraction of total time across the entire video recording that the mice entered the food bin. (**K**) The total number of seconds that each restricted female access mouse spends entering the food bin in the four hours before female access (ZT 5-9) is plotted against the number of seconds of high activity in the four hours before female access (ZT 5-9) across the entire experiment (days 0, 7, 14, 21, 28, and 35), showing the best-fit plane (R = 0.5058, p = 0.0013). (**L**) The total number of seconds that each control mouse spends entering the food bin in the four hours before feeding (ZT 5-9) is plotted against the number of seconds of high activity in the four hours before feeding (ZT 5-9) across the experiment showing the best-fit plane (R = 0.5640, p = 0.002). (**M**) The total number of seconds that each restricted female access mouse spends entering the food bin in the four hours after female access is plotted against the number of seconds of high activity in the four hours before female access showing the best-fit plane (R = 0.1565, p = 0.5723, p = 0.8201). (**N**) The total number of seconds that each control mouse spends entering the food bin in the four hours after feeding is plotted against the number of seconds of high activity in the four hours before feeding across the experiment showing the best-fit plan (R = 0.4019, p = 0.0190). For panels A–C and H–J * = p<0.05, ** = p<0.01, *** = p<0.001, Mann-Whitney Test. n = 8 at all time points. All bar graphs represent median and the upper quartile of the interquartile range.

In experiment 1, mice with restricted female access spent a similar amount of time as control mice with their noses in the food bin from ZT 5-9 except at day 28, where female-access males had a significant increase in food bin entry (Mann-Whitney Test, p<0.01) (Figure 3A). There was no difference between the time the mice with restricted female access spent with their noses in the food bin during the four hours after (ZT 10-14) female access and the amount of time the control mice spent with their noses in the food bin during the same time period (Figure 3B). Note that the amount of food bin entry time for both experimental and control groups during ZT 10-14 is drastically higher than that in ZT 5-9 as their cages have been changed just prior to video recording (ZT 10:00), leading to a burst of investigation of their environment. There was also no difference between control and restricted female access mice in total (23 hours) food bin entry time (Figure 3C). Multiple regression analysis indicates that time spent entering the food bin in the four hours prior to female access is correlated with both high activity during this time and the amount of time the mice have been treated with restricted female access (R = 0.6609, p = 0.0004) (Figure 3D). Both high activity four hours prior to access and the number of days of restricted female access have a statistically significant influence on the seconds of food bin entry during the four hours prior to access (p = 0.0328 for activity, p = 0.0091 for time). This suggests that as the number of days of restricted access increases, the experimental mice both increase their AA and anticipatory food bin entry. This relationship does not exist among controls (correlation coefficient, R = 0.3282, p = 0.0960) (Figure 3E). During the four hours following female access the number of seconds entering the food bin occurs independently of both the number of seconds of high activity in the four hours preceding female access and the number of days of restricted female access (R = 0.1208, p = 0.8201) (Figure 3F). This suggests that the AA observed in this experiment is not the consequence of self-imposed calorie restriction, as the mice that exhibit a large amount of AA are not the ones that are waiting until after female access to eat. In the control mice, there appears to be a mild inverse correlation between the seconds of high activity during ZT 5-9 and the seconds of food entry from ZT 10-14 (R = 0.328), but this is insignificant (p = 0.0960) (Figure 3G).

In Experiment 2, there was no statistically significant difference in the time mice with restricted female access spent with their noses in the food bin relative to control mice during the four hours prior to female access (ZT 5-9) (Mann-Whitney Test, p<0.01) (Figure 3H). Mice with restricted female access spent significantly more time with their noses in the food bin four hours after female access (ZT 10-14) than the control mice on days 14 and 21 (Mann-Whitney Test, p<0.01). However, this difference appears to be the result of changes in the behavior of control mice as opposed to the mice with restricted female access since the food bin entry behavior of restricted female access mice did not exhibit significant changes over time (repeated measures ANOVA with Friedman's post test) (Figure 3I). In terms of total food bin entry time, there were no differences in eating behavior except at one time point, day 7, where mice with restricted female access spent significantly less time in the food bin than controls (Mann-Whitney Test, p<0.01) (Figure 3J). As with the first experiment, mice with restricted female access that exhibit a large amount of AA also spend more time entering the food bin during the four hours prior to female access (R = 0.5058, p = 0.0013); however, high activity (p = 0.0003) makes a significant contribution but not duration of the experiment(p = 0.4300) (Figure 3K). This is similar to the trend observed in the controls (R = 0.5640, p = 0.0002), in which the number of seconds spent entering the food bin is significantly correlated with high activity (p = 0.0022) (Figure 3L). However, unlike the mice with restricted female access, duration of the experiment also causes a significant decrease in the time spent entering the food bin during ZT 5-9 (p = 0.0383). As seen with the first experiment, there is no correlation between high activity preceding restricted female access and food bin entry following female access (R = 0.1565, p = 0.5723) (Figure 3M), suggesting again that the mice that exhibit high activity are not restricting themselves to eating a large amount of food following female access. The control mice in this experiment do show a significant relationship between the days of study, high activity preceding ZT5-9, and seconds of eating from ZT 10-14 (R = 0.4019, p = 0.0190), although neither high activity (p = 0.1379) nor time (p = 0.0549) make a significant independent contribution (Figure 3N).

### Restricted Running Wheel Access

**Figure 4:**
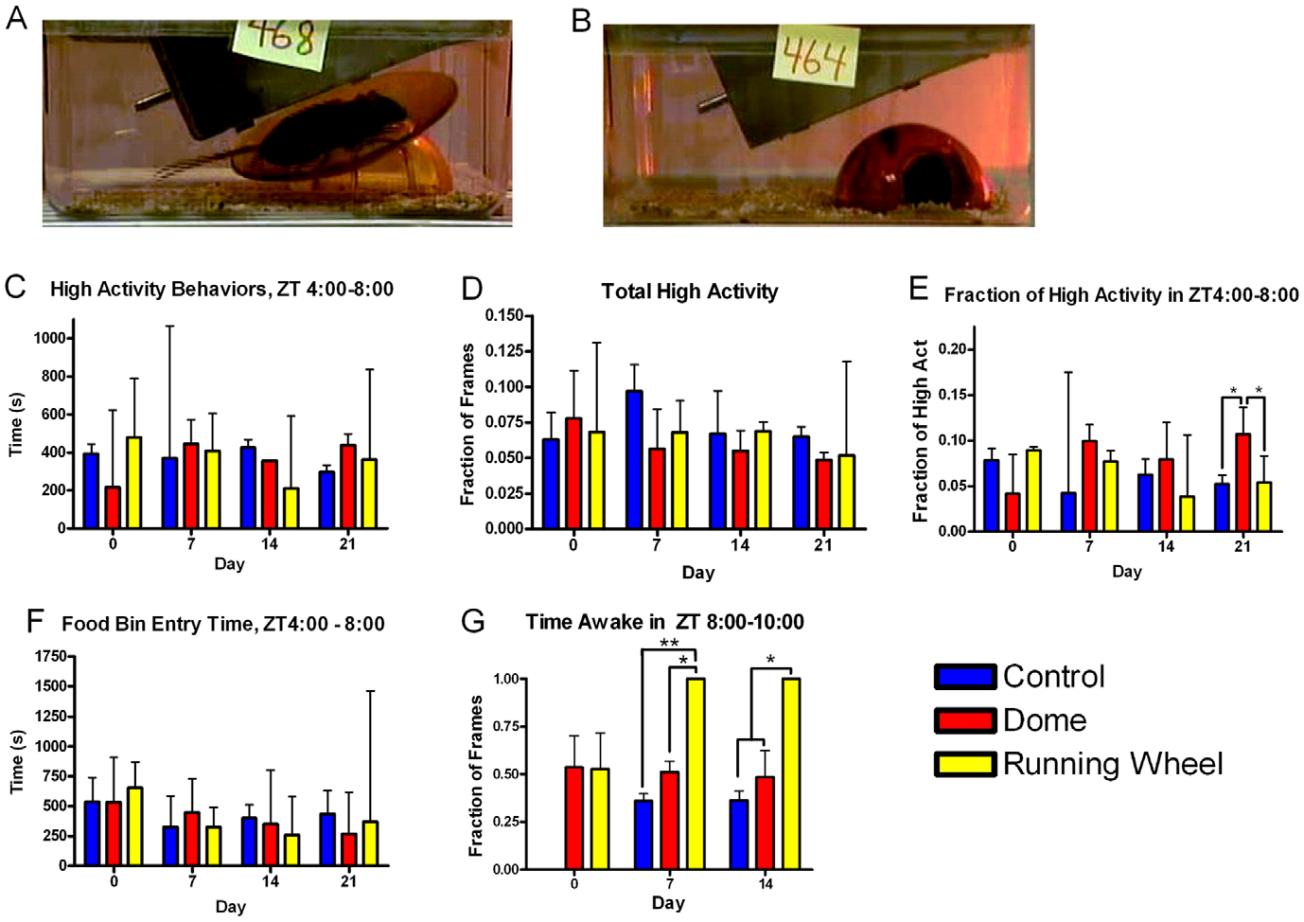
Testing the effect of restricted running wheel on anticipatory activity. (**A**) Mice have access to a “low profile” running wheel or a (**B**) a shelter dome or have their bedding slightly disturbed as another control group (not shown). (**C**) The total number of seconds of high activity during the four hours prior to receiving running wheel access. (**D**) The fraction of frames during the entire twenty-two hours of recording during which the mice exhibited the high activity behaviors. (**E**) Normalized anticipatory activity. The number of seconds during which the mice exhibited high activity behaviors in the four hours preceding wheel or dome access or bedding disturbance is divided by the total number of seconds of high activity observed in the entire twenty-two hour recording period. (**F**) Food bin entry during the four hours preceding running wheel access. (**G**) The amount of time the mouse was awake during running wheel access, dome access, or after bedding disturbance. For wheel access mice the sum of the time it spent running on the wheel, interacting with the dome or wheel, or awake but not interaction is equivalent to time awake (Kruskal-Wallis Test, * = p<0.05, ** = p<0.01) * = p<0.05, Kruskal-Wallis Test.

One possible explanation for the AA observed in mice in experiments 1 and 2 would be that these mice were disturbed and kept awake for an hour daily. To test this hypothesis in a non-social context, a new cohort (n = 17) of C57BL/6J male mice was divided into three groups: one group received running wheels (Figure 4A) for two hours daily (from ZT 8 to 10) (n = 6), one group received a shelter dome for two hours daily to control for the introduction of a large novel object (Figure 4B) (n = 5), and a third group of mice had its bedding disturbed at ZT 8 and 10 to control for the handling (n = 5). The mice were recorded for 22 hours (from ZT 10 to ZT 8 the next day) on days 0, 7, 14, and 21.

Mice that received running wheels did not exhibit any increase in activity during the four hours prior to wheel-running access relative to the mice in the control group or mice that received domes (Kruskal-Wallis Test with Dunn's Multiple Comparisons Post-Test) (Figure 4C). The total fraction of high activity during the entire twenty-two hour recording period was not significantly different between the three groups, suggesting that the two hours of running wheel or dome access does not diminish total activity during the remaining 22 hours of the day (Figure 4D). As with restricted female access experiments 1 and 2, to normalize the total activity relative to the AA, the number of seconds of high activity during the four hours preceding wheel access, dome access, or bedding disturbance was divided by the total number of seconds of high activity during the entire twenty-two hours. This normalization did not bring out any additional AA with the exception of restricted dome access mice at day 21, where they showed an increase activity during the four hours prior to receiving dome on day 21 of the experiment (Kruskal-Wallis Test with Dunn's Multiple Comparisons Post-Test) (Figure 4E, p<0.05). Restricted running wheel access mice, restricted dome access mice, and control mice had no difference in food bin entry time during the four hours prior to the access period (Figure 4F).

To quantify the amount of activity observed in mice receiving domes, running wheels, or the controls, videos recorded during at least an hour and a half of the two hours of running wheel or dome access and were manually annotated using the Caltech Behavior Annotator software. Individual frames were labeled as running on the wheel, interacting with the wheel or dome (but not running), awake but not interacting with the wheel or dome, sleeping inside the dome, or sleeping outside the dome. On days 7 and 14, mice that received access to running wheels were awake during the entire recording period, in contrast to the mice that received shelter domes or the bedding disturbance controls (Figure 4G). The data suggests that the significantly greater amount of high activity observed in mice receiving dome access on day 21 of the experiment may be due to chance, as the mice interact with the dome significantly less than they do with the running wheel.

### Restricted Social Interaction Experiment 3

From our analysis above, we concluded that the moderate AA observed in social interaction experiments 1 and 2 was neither due to self-imposed restriction of feeding behavior nor increased daily timed physical activity but it was not clear if the AA was caused by social stimulation or anticipation of a sexual interaction. To disambiguate between these possibilities, we performed a restricted social interaction experiment using female-female pairs. Female C57BL/6J mice were single housed for one week, after which they were given one hour access to a female C57BL/6 Tyr partner (phenotypically white C57BL/6J mice which can be more easily discriminated during manual annotations) from ZT 9 to 10 in their home cage for 28 days consecutively. The same female-female partners were maintained for all 28 days of the experiment.

**Figure 5:**
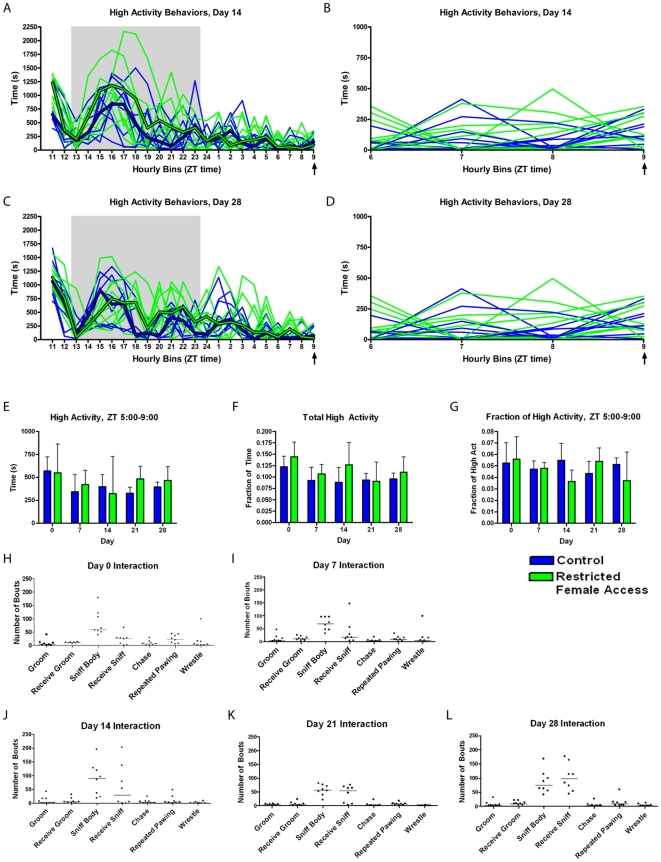
Characterizing the anticipatory activity of female mice with one hour of restricted access to a stable female partner daily (“Experiment #3”). (**A**) The number of seconds in each hourly bin of high activity behaviors (walking, jumping, rearing, and hanging) that occurred on day 14 of restricted female access. Thin weight green lines represent data from individual mice with restricted female access while blue lines represent the controls. Median values are shown in thick green and blue lines for experimental and control mice, respectively. The gray area depicts the 11 hours of lights off. (**B**) An enlarged presentation of ZT 5 to 9, the four hours preceding female access, in panel A, to show the variance in the anticipatory behavior. (**C**) The number of seconds in each hourly bin of high activity behaviors that occurred on day 21 of the experiment. (**D**) An enlarged representation of bins 6 to 9 (which includes data from ZT 5-9) in C. (**E**) The number of seconds in each hourly bin of high activity behaviors (walking, jumping, rearing, and hanging) during the last four hours prior to restricted female access. (**F**) Fraction of frames during the entire duration of the video recording (∼23 hrs) during which the mice exhibited any high activity behavior. (**G**) Normalized high activity data in the four hours preceding restricted female access. The number of seconds spent in high activity behavior during the last four hours prior to female access divided by the total number of seconds of high activity across the entire 23 hour recording. Quantifying the social interaction during the restricted female access period. The number of bouts in which each female mouse interacted with its respective female partner by grooming, being groomed, sniffing the body, being sniffed, chasing, repeated pawing, or wrestling on day 0 (**H**), day 7 (**I**), day 14 (**J**), day 21 (**K**), and day 28 (**L**). Each point on the scatter plot represents the number of instances in which an individual mouse exhibited that behavior. The solid horizontal line represents the median value.

We examined the temporal aspects of home-cage activity to determine whether female mice show an increased activity hours prior to daily female access (Figure 5A-D). There were no obvious differences between raw traces of controls and experimental mice at days 14 and 28 except for the occasional larger bursts of high activity behaviors in some restricted female access mice (Figure 5A-D). We observed that there was no difference in total seconds of high activity during the four hours prior to female access (Figure 5E), total high activity (Figure 5F), and also no difference in the normalized high activity (Figure 5G). When forty-five minutes of the one-hour daily interaction was filmed each week, we found that the females interacted on a comparable level to the males in restricted social interaction experiment #2: they exhibited between 21 and 195 bouts of sniffing as well as a diversity of other behaviors (such as grooming, chasing, repeated pawing, and wrestling) (Figure 5H-L).

**Figure 6:**
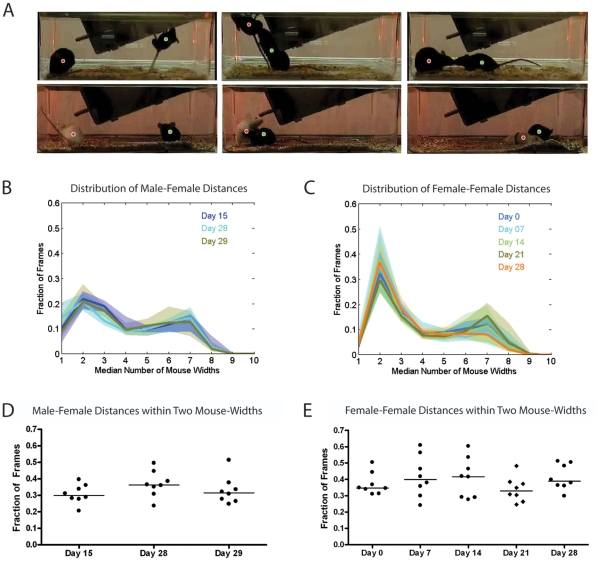
Tracking the positions of two mice during social interaction. (**A**) The tracking software is able to locate the center of masses of both mice while at different positions in the cage. Representative images of the tracker are shown, showing various social interactions between male-female pairs in the top panels and female-female pairs in the bottom panels. (**B**) The fraction of frames of a video during which the male and female mouse are within a certain number of mouse widths away from each other. The dark solid line represents the median fraction of frames the mice were within a certain distance and the shaded boundaries represent the upper and lower quartile across the individual mouse pairs for that day. Day 15 (interaction with a novel female) is shown in dark blue, day 28 (interaction with a female for the seventh day in a row) in cyan, and Day 29 (interaction with another novel female) in olive. (**C**) The fraction of frames of a video during which the two female mice are within a certain number of mouse widths away from each other. Day 0 is shown in dark blue, day 7 in cyan, day 14 in pale green, day 21 in olive, and day 28 in orange. (**D**) The fraction of frames during which the male-female mice are within two mouse widths (approximately equal to one mouse length). Each point in the scatter plot represents the value for one video (one mouse interaction at one time point). (**E**) The fraction of frames during which the female-female mice are within two mouse widths.

To more rigorously quantify the level of interaction between male-female and female-female pairs, we made use of a dual mouse automatic tracking system. Screenshots of the tracker locating the center of masses of both mice while at different positions in the cage is shown in Figure 6A. In both male-female and female-female, there was no significant variation across the days in the distribution of distances between the mice (Figure 6B-C). Both male-female and female-female pairs spent a substantial fraction of frames during the video-recording period within two body widths of each other (approximately equal to one mouse length) (Figure 6D-E), suggesting that male-female and female-female pairs spend a similar amount of time interacting.

## Discussion

In restricted social interaction experiment 1, in which virgin males were given a novel female each day, a modest AA when we normalized for total activity was evident from two weeks of restricted female access until the experiment was terminated at four weeks (Figure 1). In social interaction experiment 2, in which males were initially cohabitated with females and then given daily access to stable partners, males displayed mild AA at days 7 and 14 but not at the later measurements at days 21, 28, and 35 (Figure 2). The males appeared to interact slightly more with novel females than familiar ones, with two out of eight males increasing mounting behavior and a small increase in median investigation behavior (Figure 2J), although not enough individuals were used to determine if this difference was significant and not enough time points were recorded to determine if the duration of the experiment was a contributing factor. The automatic tracker results also indicated that there was no substantive difference between the distributions of distances between the male-female pairs across the three time points during which the interaction may occur (Figure 6B). Sexual experience may explain the differences in the timing of AA observed between the two experiments. The late onset of anticipation in the first social experiment, which was seen most strongly at day 28 (Figure 1E), may be the result of sexual inexperience, since mice are known to increase their interest in female urine after they have become sexually experienced [20]. In experiment #1 the males may have been learning courtship and building a positive association with successful copulation while in experiment #2 the males, which had been housed with females continuously for one week prior to the beginning of the experiment, showed AA earlier. Although there is a notable trend toward increased AA at days 21, 28, and 35 (Figure 2G), it is not clear why the males in experiment #2 stopped showing increased AA for female access.

A similar study of restricted socio-sexual interactions in rats reported that scheduled mating at ZT6 significantly increased food intake during the following 6 h, and that the number of rats showing AA to daily mate access significantly decreased when post-coital feeding was prevented (Landry et al, unpublished results). Thus, we examined our data set to determine if eating behavior was related to restricted female access. In social experiment 1, there was a correlation between anticipatory food bin entry and AA (Figure 3D) but there was no correlation between post-interaction food bin entry and high activity (Figure 3F), demonstrating that the restricted female access mice were entering the food bin slightly more in anticipation of female access but not eating more afterwards. In Social Experiment 2, increased AA is correlated with increased food bin entry time prior to interaction (Figure 3K), while post-interaction food bin entry time was not correlated with the magnitude of high activity (Figure 3M). Although the experimental mice did spend significantly more time entering the food bin in the four hours following interaction than the control mice did on days 14 and 21 (Figure 3I), this was not significantly different from the amount of time they spent eating during the same time interval than on day -7, prior to any female access. Mice with restricted female access also demonstrated AA on day 7, despite having no difference in food bin entry time. There is no correlation between the time spent entering the food bin following female interaction and the amount of AA preceding female interaction (Figure 3M). This data suggests that the increase in high activity during the four hours prior to female interaction is independent of feeding behavior.

When given restricted access to running wheels, mice are awake during the entire time they have running wheel access (Figure 4G). Previous research demonstrates that blocking opiod receptors decreases wheel running in mice that are first being exposed to running wheels, suggesting that reward mechanisms are involved in wheel running [21]. Surprisingly, one study has shown that wheel running is rewarding enough to outcompete conditioned place preference for morphine in rats [22]. However, despite this strong daily rewarding stimulus, mice with restricted running wheel access are not significantly more active during the anticipatory period than either mice with restricted dome access or the controls (Figure 4C). These results indicate that being consistently awake and active for two hours a day is not by itself sufficient to induce AA. The absence of AA observed in social experiment 3 (female mice receiving once daily access to a companion female), despite the substantial amount of social interaction, suggests that social stimulus alone is also not sufficient to produce AA without sexual interaction. Although previous literature has described physiological and behavioral anticipation in response to a scheduled non-physical scheduled contact with another aggressive mouse (4 consecutive days of interaction in a divided cage) [23], the differences between our results and those described in the literature may be the result of the differences in gender (males may be more likely to anticipate social interactions than females), or the need for an extremely stressful or emotional social stimulus.

One of the more interesting results of this experiment was the fact that robust AA was not consistent within animals, as it suggests that AA need not be expressed daily in order to persist (Figures 1H and 2K). One factor that may influence the fluctuation in AA that we observed could arise from copulation, as previous studies have shown that ejaculation ends a male's interests in sexual behavior for at least twenty-four hours, although males have been known to copulate within several hours [24]. Another possible contributor to the variable response to daily restricted female access is that the females were not always in estrus, and thus unlikely to be willing to mate for many of the restricted access periods. Pregnancy-induced anestrus may have lead to a lack of female receptivity as females were used continuously for one week in restricted social interaction experiments 1 and 2, but only 2 out of 12 tested actually gave litters (data not shown) suggesting a low rate of successful copulation. Also, because the males in the first experiment were housed in groups of four until two or three days prior to restricted female access, many of males used in this study were likely of subordinate social status. Previous research suggests that if a subordinate male is cohabitated with a dominant male, the subordinate male decreases investigation and sexual interaction with a female as the period of cohabitation increases, despite the fact that the dominant male is not present for these interactions [25]. Differences in reproductive success between subordinate and dominant males were observed to become negligible after one to three weeks of single-housing [25]. For restricted social interaction experiment #2, the males were not housed with other males for at least nine days prior to the restricted female access. This may account differences in the timing of the behavioral response to restricted female access that we observed between restricted social interaction experiments 1 and 2. A further iteration of this experiment should single house the male mice from weaning to fully establish dominant status and present to them at least one hormone primed (receptive) female mouse at the same time each day.

Although our results suggest that non-sexual social stimulus alone is not enough to induce AA in females, this result can be tested in male mice with restricted access to females in estrus in comparison to restricted access to ovariectomized females or to those not in estrus. In addition, future experiments can be improved by allowing males to be cohabitated with females for a week prior to restricted access, thereby allowing them to both gain sexual experience and recover from their previous subordinate status. To account for the loss of interest in mating post-ejaculation and other factors that influence AA caused restricted female access, it will be important in future studies to both record activity and social interaction with a higher sampling rate, ideally every day. The time of day for sexual and/or social interaction is another variable that could be modified, as rats show a much higher interest in copulation in the early part of the dark cycle [26], which unfortunately would be a difficult time to detect AA due to the normal night time activity peak. Finally, it would also be of interest to determine whether sexual/social AA is able to persist after a one-day withdrawal of the stimulus. This has been observed previously in anticipation of other stimuli such as food [27], palatable meals [14]–[15], methamphetamine [23], and encounters with aggressive males [28]. Recently, we even observed a reaction to withdrawal of a daily stimulus (a daily palatable meal of high fat diet) in female C57BL/6J mice that did not show AA for 28 consecutive days of meal delivery, highlighting the potential importance of withdrawal experiments even when no behavioral response is observed [29].

Taken together, these results and possible future iterations of social/sexual anticipatory conditions suggest that limited mate access causes mild AA, albeit this is far less robust than that observed with restricted food access. Importantly, these results suggest that food is not a wholly unique zeitgeber for the anticipatory arousal system, a question recently phrased by Webb *et al.* [16], thereby extending the possible models of anticipation beyond restricted feeding.

## Materials and Methods

### Mouse Strains and Husbandry Conditions

All experiments were approved by the Caltech Animal Care and Use Committee. Male and female C57BL/6 mice ranging from 10–12 weeks of age were ordered from Jackson Labs and single housed for forty-eight to seventy-two hours prior to initiating each experiment. All mice were allowed *ad libitum* access to LabDiet Laboratory Rodent Diet 5001 and water and were on a 13 hours of light 11 hours of dark cycle.

### Video Based Behavioral Analysis

The videos of singly housed mice in the home cage were analyzed by an automated behavior recognition system, HomeCageScan 3.0 [18]–[19], and data was output into twenty-three hour bins to facilitate understanding of the temporal structure of activity. Dim red lighting (Philips 25 watt “party and deco” bulbs) was used to record during the 11 hour dark cycle. Manual annotations of social/sexual interactions and wheel running activity were done manually using the Caltech Behavior Annotator program, a software tool created in MATLAB designed to facilitate the rapid manual annotation of video sequences. We used an automated computer vision based tracking system to measure the distance between pairs of mice in social experiments #2 and #3. The system attempts to calculate the center of mass of each of the two mice in recorded video frames. Visual inspection of a number of videos showed that the tracking results were generally reasonable. We also performed a more systematic analysis of tracking performance, classifying 1000 frames randomly selected from 24 male/female videos and likewise 1000 frames randomly selected from 37 female/female videos. The tracking system performed better for black mice, hence performance was higher for the male/female videos which contained two black mice than for the female/female videos which contained one black and one white mouse. A technician manually classified the tracking results in each of the 2000 frames via visual inspection. The center of mass for each mouse can be visualized as a colored dot (see Figure 6A); the technician classified each frame as "good" (one dot on each mouse), "acceptable" (mice overlapped, dots near but not on center of mass), and "bad" (either dot outside the extent of one of the two mice). In the female/female (black/white) videos, the technician annotated 5.6% of frames as "bad", 1.6% as "acceptable", and the remaining 92.8% as "good". In the male/female (black/black) videos, 1.6% of frames were labeled "bad", 1.2% as "acceptable", and the remaining 97.2% as "good". Since "acceptable" frames do not adversely affect our analysis, overall the tracker performed well in 94.4% and 98.4% of frames in the female/female and male/female videos respectively. See Figure 6A for examples of the tracker output on representative frames. Mouse width was equivalent to 35 pixels, which was the mean width taken from 1393 ellipses manually drawn over the female access.

### Social Experiment 1

Twelve C57BL/6J male mice were divided into two groups: one group of six mice received restricted access to a C57BL/6J female, the other group of six mice was used to control for handling conditions. At the start of each week, one virgin female was deposited into the cage of each experimental male mouse at ZT 9 and removed at ZT 10. Each of the six females were given to a novel male partner for the next six days. On the seventh day of each week, the male mice were recorded for twenty-three hours prior to receiving the same female from the first day of the week. The female mice were returned to group housing (4 female mice per cage) at the end of each hour, and sacrificed after seven days of interaction. To control for handling conditions, control mice were moved to the hood and had their food rack lifted at ZT 9 and ZT 10. All male mice were video recorded in their home cages at days 0, 7, 14, 21, and 28 of this treatment.

### Social Experiment 2

Sixteen C57BL/6J male mice were cohabitated with a female partner for seven days prior to the experiment. Starting on day 0, the male mice were divided into two groups, one group of eight experimental mice that received restricted access to a female and another group of eight mice that was used to control for handling condition. A novel virgin female was placed in the cage of each experimental mouse at ZT 9 and removed and returned to group housing (4 female mice per cage) at ZT 10:00. Each experimental male received access to the same partner from ZT 9 to ZT 10 each day for seven days. The mice were video-recorded from ZT 10 until ZT 9 the following day (for 23 hours) every seven days. At the end of each recording, the female was sacrificed and the male received a new virgin female partner in its cage for one hour for seven days. To control for handling conditions, control mice were moved to the hood and had their food wire lifted at ZT 9 and ZT 10:00. All male mice were recorded for twenty-three hours on day -7 (prior to receiving a female partner), 0, 7, 14, 21, 28, and 35. The one hour interaction between mice were recorded on day 15, 28, and 29 for quantifying social interaction. Interaction behaviors were defined from the perspective of the male mouse and labeled as either sniff body, chase, thrust, or mount.

### Social Experiment 3

Sixteen C57BL/6 female mice, 10 weeks old, were singly housed for six to eight days prior to their first restricted social interaction with a C57BL/6J- Tyr(c-2J)/J female (17-21 weeks old). Starting on day 0, the female mice were divided into two groups, one group of eight experimental mice that received restricted access to a stable female partner for one hour daily from ZT 9 to ZT 10 for the entire duration of the experiment and another group of eight mice that was used as a control and handled twice daily at the same times as the experimental mice. Female C57BL/6- Tyr(c-2J)/J (which have a white coat color caused by a single gene mutation in an otherwise C57BL/6 genetic background) were single housed at all times except when interacting with C57BL/6J females. The C57BL/6J female mice were video-recorded from ZT 10 until ZT 9 (for 23 hours) weekly (Day 0, 7, 14, 21, and 28). The one hour of social interaction (ZT 9-10) was also recorded weekly for quantifying of social interaction. Interaction behaviors were defined from the perspective of the C57BL/6 (black mice), and labeled as one of the following: groom (black mouse grooming white mouse), receive groom (white mouse grooming black mouse), sniff body (black mouse with its nose in contact with the white mouse), receive sniff (white mouse with its nose in contact with the black mouse), chase (any instance where the black mouse approached the white mouse at a high velocity), repeated pawing (any time when the white mouse was moving and the black mouse touched its paws with it for more than three times), of wrestling (any instance with a large amount of interaction and movement and the dominant mouse was unidentifiable).

### Statistical Analysis

Nonparametric tests were chosen for analysis of behavioral data as activity patterns did not follow a normal distribution. Statistical significance tests and multiple regressions were generated using GraphPad Instat. Figures 3D through 3G and 3K through 3L were generated using the MESH and STEM3 functions in MATLAB.

### Restricted Running Wheel Access Experiment

Sixteen male mice received access to a low-profile running wheel for twenty-four hours. The next day, the mice were divided into three groups: one group of six mice received access to a low-profile running wheel from ZT 8 to ZT 10, one group of five mice received access to a dome from ZT 8 to ZT 10 (to control for the presence of a large novel object), and one group of five mice had a corner of their bedding slightly disturbed at ZT 8 and ZT 10. The mice were recorded on day 0, 7, 14 from ZT 10 until ZT 8 the following day and on day 21 from ZT 10 until ZT 10 the next day. The mice did not receive dome or wheel access on day 21 of the experiment.

On days 0, 7, and 14, the mice were video-recorded during at least one and a half hours of the two hours of wheel access and dome access. Controls were recorded on day 7 and day 14 during this time. Caltech Behavior Annotator software was used to manually the behavior as either running on the wheel, interacting with the dome or the wheel, awake but not interacting, sleeping inside the wheel or dome, or sleeping outside the wheel or dome. Running was defined as having all four feet on the wheel and moving. Interaction was defined as any contact with the wheel or dome or any time the mouse and the dome or wheel appeared to overlap from the camera's point of view. Any time the mouse was awake but not either running or interacting with the wheel was considered awake but not interacting. The amount of interaction for one mouse in the running wheel group was excluded from day 7, as his wheel was not functional on that day.
